# Medical Care and Attention to the Indigent Sick

**Published:** 1880-06

**Authors:** 


### EDITORIAL DEPARTMENT.

"If thou hast truth to utter, speak;
And leave the rest to God."

MEDICAL CARE AND ATTENTION TO THE INDIGENT SICK.—The *Free Dispensary* system, as it exists in our cities, has been, and is still attracting a large share of attention among those who feel desirous of doing all that is attainable for the relief and comfort of the sick and helpless in indigent circumstances. A large and rapidly growing city like Baltimore, should have a CITY HOSPITAL, centrally located, in a salubrious and airy space, with all the modern appurtenances relating to such an institution; always in readiness, with ambulances, or other facilities, for conveying sick or wounded persons requiring medical or surgical treatment, to its wards.

Such an institution with skillful and intelligent medical men, well trained nurses and proper discipline, would add greatly to the preservation of human life and the mitigation of human suffering in our midst. With a City Hospital equipped in this manner, and the additional facilities offered by Bay View and the charity department of Johns Hopkins' Hospital, it would only remain to make provision for the indigent who could not be, and in some cases, ought not to be, removed from their homes. These latter could be cared for in a suitable manner by *districting* the city and appointing a *Physician to each district*, to visit, when necessary, at their homes, and prescribe for *all* persons unable to pay a doctor's bill, in their respective districts. Four or more, drug stores, if necessary, owned by the City, should be established in appropriate localities, and these alone should supply the medicines ordered by the City Physicians. It is hardly necessary to add, that to make the proposed plan work efficiently, the salary of the district physicians should be such as to secure medical men of intelligence and experience in the profession, for these positions.

Such a plan as the one proposed, would assuredly save many lives that would otherwise be lost, and contribute vastly to the relief of the thousand ills that flesh is heir to. By this means the obnoxious *free dispensary* system would be done away with and medical affairs permitted to flow again in their old and proper channels. Public attention has been awakened and it only requires that the impression already made be kept alive by frequent, but judicious suggestions, in order to bring about soon the result so much desired in the proper disposal of medical help and assistance to the indigent and needy in our midst.

As we are to have the poor always with us, economy may be combined with justice, and mercy to them, if a little forethought and prudence are allowed a place in our philanthropy.

